# Nafion/Surface Modified Ceria Hybrid Membranes for Fuel Cell Application

**DOI:** 10.3390/polym13152513

**Published:** 2021-07-30

**Authors:** Polina A. Yurova, Viktoria R. Malakhova, Ekaterina V. Gerasimova, Irina A. Stenina, Andrey B. Yaroslavtsev

**Affiliations:** 1 Kurnakov Institute of General and Inorganic Chemistry of the Russian Academy of Sciences, Leninsky Prospect 31, 119991 Moscow, Russia; polina31415@mail.ru (P.A.Y.); vika.malakhova.0207@gmail.com (V.R.M.); stenina@igic.ras.ru (I.A.S.); 2 Basic Department of Inorganic Chemistry and Materials Science, National Research University Higher School of Economics, ul. Myasnitskaya 20, 101000 Moscow, Russia; 3 Institute of Problems of Chemical Physics of the Russian Academy of Sciences, prospect Academician Semenov 1, 142432 Chernogolovka, Moscow region, Russia; krizhi@gmail.com

**Keywords:** Nafion, hybrid membranes, surface modification, ceria, ionic conductivity, fuel cell

## Abstract

Low chemical durability of proton exchange membranes is one the main factors limiting their lifetime in fuel cells. Ceria nanoparticles are the most common free radical scavengers. In this work, hybrid membranes based on Nafion-117 membrane and sulfonic or phosphoric acid functionalized ceria synthesized from various precursors were prepared by the in situ method for the first time. Ceria introduction led to a slight decrease in conductivity of hybrid membranes in contact with water. At the same time, conductivity of membranes containing sulfonic acid modified ceria exceeded that of the pristine Nafion-117 membrane at 30% relative humidity (RH). Hydrogen permeability decreased for composite membranes with ceria synthesized from cerium (III) nitrate, which correlates with their water uptake. In hydrogen-air fuel cells, membrane electrode assembly fabricated with the hybrid membrane containing ceria synthesized from cerium (IV) sulfate exhibited a peak power density of 433 mW/cm^2^ at a current density of 1080 mA/cm^2^, while operating at 60 °C and 70% RH. It was 1.5 times higher than for the pristine Nafion-117 membrane (287 mW/cm^2^ at a current density of 714 mA/cm^2^).

## 1. Introduction

In recent years, there has been an increasing interest in the modification of cation-exchange membranes by introducing inorganic oxides into the membrane pores and the channels system. Most of the works in this area are devoted to silica and zirconia [1,2,3,4,5,6], while far too little attention has been paid to ceria [7,8,9]

During operation of low-temperature fuel cells (FCs), hydrogen peroxide and other reactive oxygen species (hydroxyl radicals, superoxide radicals) are generated in a number of side electrochemical reactions. These radicals cause proton-exchange membranes to degrade and decrease FC power [10,11,12,13]. Cerium ions can interact with reactive oxygen species due to the reversible redox reaction Ce^4+^ ↔ Ce^3+^ [14,15,16,17]. Cerium ions can be introduced into the membrane matrix by ion-exchange [18,19] or by casting of the membrane solution with previously prepared CeO_2_ nanoparticles [20,21,22]. The introduction of ceria can also reduce the rate of membrane degradation [19,22,23,24,25]. Velayutham at al. showed that Nafion membranes doped with 1 wt% CeO_2_ exhibited a decrease in methanol permeability and an increase in conductivity and power density of a methanol fuel cell based on them [26]. However, in many cases, ceria introduction has the opposite effect (proton transport rate, conductivity of hybrid membrane and/or fuel cell performance decrease, at best, does not change) [19,25,27,28,29,30].

This is most likely due to the large radius of cerium cations determining the basic nature of ceria surface. Therefore, CeO_2_ nanoparticles should form salt bridges with functional groups of membrane pore walls resulting in their crosslinking. This should lead to the exclusion of a part of functional groups from proton transfer and to a decrease in the membrane water uptake as in the case of hybrid membranes with zirconia [31]. A possible way to mitigate the conductivity decrease can be an increase in the acidity of ceria surface by its functionalization with acid groups [32,33]. In some cases, such a modification allows one to increase the conductivity, water uptake, and the selectivity of the prepared hybrid membranes [32,34,35]. Most of the works are devoted to the modification of membranes by acid functionalized silica, titania, or zirconia [32,35,36,37,38,39,40]. To the best of our knowledge, there is no information on composite membranes doped by ceria with a surface modified by acid groups. At the same time, ceria functionalized by sulfonic or phosphoric acid groups have demonstrated an increase in conductivity by almost two orders of magnitude [41]. This allows us to consider such materials as promising dopants for proton-exchange membranes. 

In the present work, hybrid membranes based on Nafion-117 and ceria synthesized from various precursors and functionalized by sulfonic or phosphoric acid groups were prepared for the first time. The proton conductivity and hydrogen permeability of composite membranes as a function of temperature and relative humidity was investigated. In addition, membrane electrode assemblies (MEAs) containing studied membranes were tested.

## 2. Materials and Methods

The modification of homogeneous perfluorinated sulfonic acid membranes Nafion-117 (thickness of 190–200 µm, Du Pont de Nemours, Wilmington, DE, USA) with ceria was carried out by the in situ method. The following solutions were used as ceria precursors: 0.01 M Ce(NO_3_)_3_ (Sigma-Aldrich, St. Louis, MO, USA), 0.3 M (NH_4_)_2_Ce(NO_3_)_6_ (Sigma-Aldrich, St. Louis, MO, USA), 0.05 M Ce(SO_4_)_2_ (Sigma-Aldrich, St. Louis, MO, USA), and 0.05 M (NH_4_)_4_Ce(SO_4_)_4_ (Sigma-Aldrich, St. Louis, MO, USA).

Before the modification, Nafion-117 membranes were conditioned by sequential boiling in 3% H_2_O_2_ solution, 5% HCl solution, and deionized water for 2 h. Then, they were placed in a ceria precursor solution for 1 h, treated with a diluted aqueous ammonia solution and re-conditioned by keeping in 5% HCl solution and deionized water at 25 °C for 1 h. Hybrid membranes prepared using Ce(NO_3_)_3_ and (NH_4_)_2_Ce(NO_3_)_6_ solutions were treated with 0.2 or 1 M sulfuric acid or phosphoric acid for modification of the surface of ceria in membrane pores and channels with sulfonic or phosphoric acid groups, respectively. Since oxides of polyvalent elements prepared by precipitation from aqueous precursor solutions are usually nanocrystalline, their surface contains a significant amount of ions sorbed from solutions [42,43]. It can be assumed that ceria precipitation from cerium sulfate solution will lead to a modification of the surface of obtained oxide by sulfonic acid groups. Thus, no additional composite membrane treatment was performed when Ce(SO_4_)_2_ and (NH_4_)_4_Ce(SO_4_)_4_ solutions were used to synthesize ceria. The designations of the prepared hybrid membranes, the precursors of ceria, and the acid used to modify its surface are listed in Table 1.

The cross-sectional morphology of the modified Nafion-117 membranes was studied using a Carl Zeiss NVision 40 scanning electron microscope (SEM) (Carl Zeiss Group, Oberkochen, Germany) equipped with an Oxford X-Max energy dispersive X-ray spectrometry detector (Oxford Instruments, Abingdon-on-Thames, UK). IR-spectra were registered in a Nicolet iS5 FTIR spectrometer (Thermo Fisher Scientific, Waltham, MA, USA) employing a diamond Specac Quest ATR add-on (attenuated total reflection mode). X-ray diffraction patterns were recorded using a Rigaku D/MAX 2200 diffractometer (Rigaku, Tokyo, Japan), CuK_α_ radiation. Water uptake was determined on a Netzsch TG 209 F1 thermal balance (NETZSCH, Selb, Germany) in platinum crucibles in the temperature range 25–200 °C under argon atmosphere. To determine the dopant content, composite membranes were annealed at 800 °C in air for 0.5 h. The ion exchange capacity (IEC) of membranes was determined by acid-base titration [44]. The IEC values are given per 1 g of the dry membrane. The conductivity was measured using an impedance analyzer Z1500 PRO Elins (10 Hz–1.5 MHz, Elins LLC, Chernogolovka, Russia) by a two-contact method on symmetric cells with graphite electrodes under an ac signal amplitude of 80 mV. The measurements were carried out in deionized water and at 30% relative humidity (RH) in the temperature range of 25–80 °C. The ionic conductivity at each temperature was obtained using a Nyquist plot from complex impedance analysis. Electronic conductivity was measured at direct current. Hydrogen permeability was studied using a Crystallux-4000M gas chromatograph (RPC Meta-chrom, Co. Ltd, Yoshkar-Ola, Russia) according to the procedure described elsewhere [44]. Mechanical analysis was performed at 25 °C and 32% RH using a Tinius Olsen H5KT universal testing machine (Tinius Olsen, Horsham, PA, USA) with a Tinius Olsen 100R extensometer with 5 mm/min strain rate. Before stress-strain measurements, membrane samples (rectangular specimens of 100 mm length and 10 mm width) were pre-equilibrated at 25 °C and 32% RH. Each measurement was repeated 5 times (five identical samples of each membrane were used).

The hybrid membranes were aged ex situ using the Fenton reagent (20 ppm Fe(II) in 30 wt% in H_2_O_2_) prepared immediately before use. A membrane sample (40–50 mg) was stored in the Fenton reagent (20 mL) at 75 °C for 10 h. The fluoride ion concentration in the Fenton reagent after membrane ageing was measured using a Mettler Toledo fluoride ion selective electrode. The fluoride ion selective electrode was calibrated using a series of standard solutions. To keep the pH in the range of 5–7 and prevent complexation of fluoride ions by iron, a total ionic strength adjustment buffer was added to the Fenton reagent solution after membrane ageing.

Membrane electrode assembly (MEA) fabricated with Nafion 117 and NC4(S) membranes were studied in the fuel cell with a working area of 1 cm^2^ (Electrochem, Inc., Woburn, MA, USA). Catalyst ink (weight ratio Nafion®/C is of 0.7) was sprayed onto a surface of a Freudenberg H23C8 gas diffusion layer using a Prism BT setup (USI, Haverhill, MA, USA). Loading of the Pt/C catalyst in the MEA per Pt content was 1.0 mg/cm^2^ (49 wt% Pt/carbon black, Inenergy LLC, Moscow, Russia). The MEAs were fabricated by hot pressing at 130 °C and 80 atm for 3 min. The assembled MEA was tested using a Greenlight Innovation G40 test station at 25 and 60 °C and 100 and 70% RH of the supplied gases, respectively. Gas flow rate was 0.1 L/min for hydrogen and 0.4 L/min for air. Chronoamperograms were measured using an Autolab PGSTAT302 N (Metrohm AG, Utrecht, The Netherlands). The currents changing with a rate < 1%/min were assumed to be steady-state. Before measurements, FCs were kept at 400 mV for 3–8 h to achieve the steady-state performance and optimization of three-phase boundaries.

## 3. Results and Discussion

Dopant content, ion-exchange capacity, and water uptake of the prepared composite membranes are shown in Table 2. The dopant content was slightly higher in hybrid membranes prepared from a precursor containing cerium in the cationic form (Ce(NO_3_)_3_ or Ce(SO_4_)_2_ salt) than that for samples obtained from precursors containing cerium in the anionic form ((NH_4_)_2_Ce(NO_3_)_6_ or (NH_4_)_4_Ce(SO_4_)_4_ salt), despite the fact that the concentration of Ce(NO_3_)_3_ solution was 30 times lower than the (NH_4_)_2_Ce(NO_3_)_6_ concentration. This effect was also clearly seen for membranes prepared from the solutions of cerium sulfate and cerium-ammonium sulfate of the same concentration. This is explained by the fact that cation-exchange membranes adsorb cations to a much greater extent than anions. When ceria precipitated from Ce(NO_3_)_3_ was treated with NaH_2_PO_4_ or NaHSO_4_ solution, cerium(III) phosphate or cerium(III) sulfate was formed [41]. Similar processes can occur in the NC3 hybrid membrane treated with dilute phosphoric or sulfuric acid. The dopant content was significantly higher in the NC3_0.2P membrane than in the NC3 membrane due to CePO_4_ formation, the molar mass of which is higher than the molar mass of CeO_2_. Similarly, treatment of the NC3 membrane with 0.2 M H_2_SO_4_ solution led to Ce_2_(SO_4_)_3_ formation and the dopant content remained almost unchanged. With increasing the concentration of acids (up to 1 M), the dopant (ceria) was partially dissolved, and its content in the composite membranes decreased. Indeed, when freshly precipitated ceria was treated with 1 M H_2_SO_4_, its partial dissolution was observed, while a CeO_2_ sample, kept in air for a month, was much less prone to dissolution when treated with acids.

**Figure 1:**
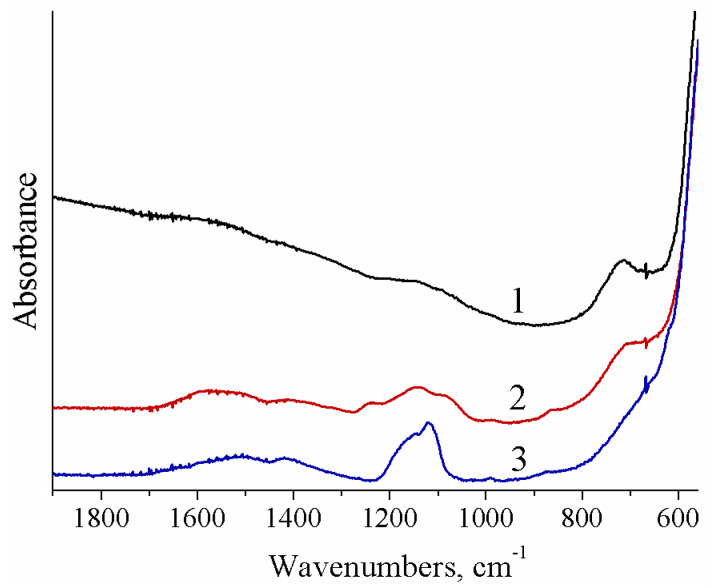
IR spectra of residues of the NC3 (1), NC3_0.2P (2), and NC3_1S (3) hybrid membranes after annealing at 800 °C.

According to the X-ray diffraction data, the residues of all composite membranes after annealing at 800 °C are represented by CeO_2_ without any impurity of cerium phosphate or cerium sulfate (Figure S1). At the same time, the IR spectra of the membrane residues showed rather intense bands in the region of 1000–1200 cm^–1^, corresponding to the vibrations of the SO_4_^−^ and PO_4_^−^ groups (Figure 1). This indicates that only the surface of ceria in the membrane pores and channels was modified. It can be assumed that sulfonic acid groups can also be grafted onto the ceria surface during annealing from SO_3_–groups of the Nafion-117 membrane. However, there are no bands in the region of SO_3_–group vibrations in the IR spectrum of the NC3 membrane residue (Figure 1). It should be noted that IR spectra of composite sulfonic acid membranes are not informative when studying the modification with dopants containing sulfonic acid or phosphate groups, since in the region 1000–1100 cm^−1^, there is an intense band corresponding to the vibrations of SO_3_–groups of the Nafion-117 membrane.

The ion-exchange capacity of all hybrid membranes was lower than that of the pristine Nafion-117 membrane (Table 2). This may be due to the fact that in hybrid membranes, some sulfonic acid groups are bound by strong salt bridges with cerium ions on the surface of CeO_2_ nanoparticles and do not take part in other ion-exchange reactions. Similar interactions of acetic acid with ceria surface were described elsewhere [45]. At the same time, the ion-exchange capacity for most composite membranes was almost the same.

The water uptake of the NC3 membrane did not differ from that of the pristine Nafion-117 membrane (Table 2). The treatment of ceria in membrane pores with phosphoric acid did not lead to noticeable changes in the water uptake of the composite membrane, while the treatment with sulfuric acid resulted in its increase. The water uptake of all hybrid membranes prepared from precursors containing Ce(IV) was slightly higher than the water uptake of the Nafion 117 membrane, and for all hybrid membranes it increased with an increase in the concentration of the acid used for the membrane treatment. The water uptake of all membranes kept at 30% relative humidity (RH) for 7 days decreased more than four times. Moreover, for the composite membranes, this drop was more pronounced than for the pristine Nafion-117 membrane. At the same time, it is surprising that the NC4(S) membrane, which exhibited the best conductivity at 30% RH, had one of the lowest water uptake at this relative humidity.

**Figure 2:**
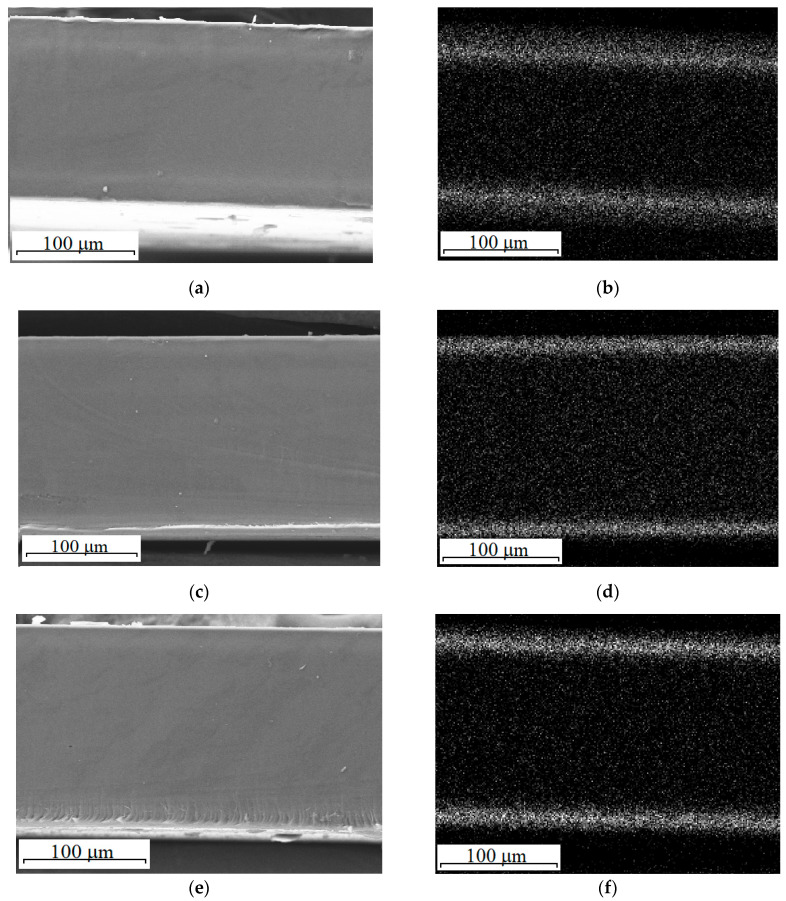

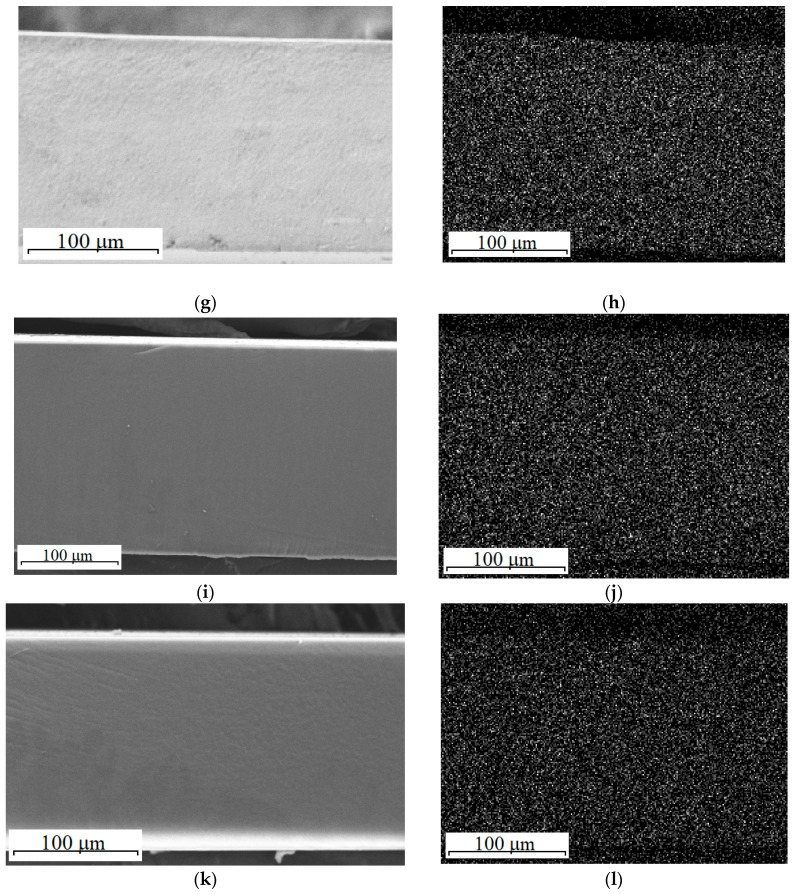
The cross-section SEM images (**a**,**c**,**e**,**g**,**i**,**k**) and EDS cerium mappings (**b**,**d**,**f**,**h**,**j**,**l**) of the NC3 (**a**,**b**), NC3_1HP (**c**,**d**), NC3_1HS (**e**,**f**), NC4 (**g**,**h**), NC4(S) (**i**,**j**), and NC4(NS) (**k**,**l**) composite membranes.

Figure 2 shows the cross-section SEM images and corresponding EDS cerium mappings of the composite membranes. For all the composite membranes of the NC4 series, the distribution of ceria over the membrane thickness was uniform, while in the composite membranes of the NC3 series, the dopant was concentrated in the near-surface region (Figure 2). The reason is that in the case of the Ce(NO_3_)_3_ or Ce(SO_4_)_2_ precursor use, cerium cations, as described above, form strong bonds with the sulfonic acid groups of the Nafion-117 membrane, which prevents their further movement into the membrane. On the contrary, the anionic forms of cerium (Ce(NO_3_)_6_^2^^−^ or Ce(SO_4_)_4_^4^^−^) are rather easily transferred through the electrically neutral solution located in the pore centers, which leads to the uniform distribution of cerium over the hybrid membrane thickness. However, due to the Donnan exclusion, the anion concentration is low in the cation-exchange membrane, which is confirmed by the low dopant content in the composite membranes prepared using precursors containing cerium in the anionic form (Table 2). On the other hand, cerium sulfate is stable in aqueous solutions only at low pH values. Its hydrolysis occurs even when the membrane is kept in the Ce(SO_4_)_2_ solution (without ammonia treatment), as evidenced by the solution turbidity. Thus, cerium ions can transfer into the membrane as part of hydroxosulfocomplexes with a low charge, which leads to their uniform distribution over the membrane thickness.

**Figure 3:**
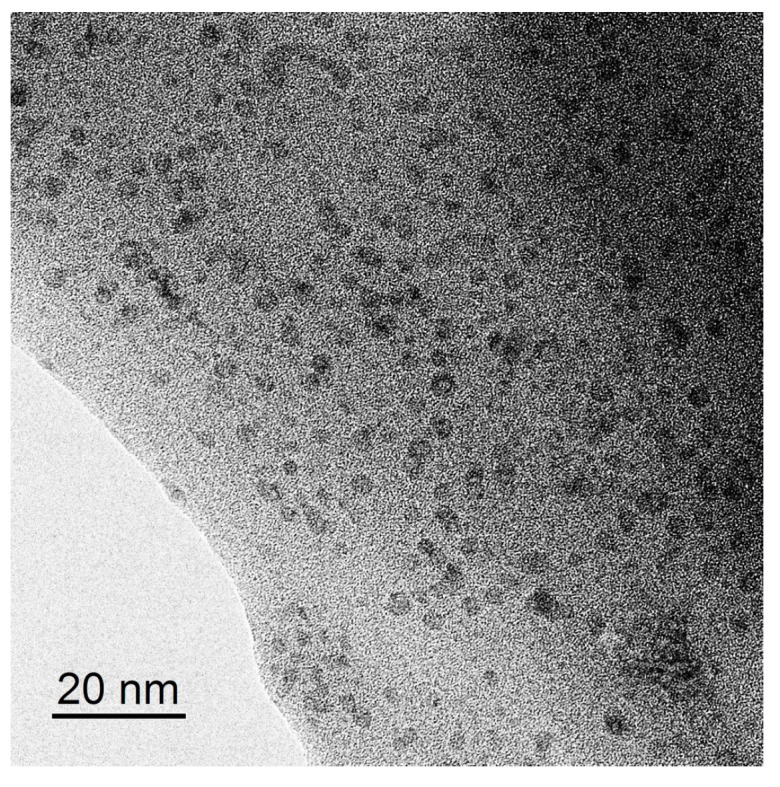
TEM image of the NC3-0.2S hybrid membrane.

SEM resolution is not sufficient to see particles incorporated into the pores of homogeneous Nafion membranes by the in situ method. Both the pristine and hybrid membranes looked like homogeneous polymer films on SEM images (Figure 2). Nanoparticles were visible on TEM images, and one of them for the NC3_0.2S hybrid membrane is given in Figure 3.

The Nyquist plots for the of the NC4(S) composite membrane are shown in Figure S2. The intercept of the Nyquist curves in the high-frequency region with the real resistance axis (Z’) represents the through-plane proton transport resistance of the membrane. Such impedance spectra are typical for all membranes under study. 

**Figure 4:**
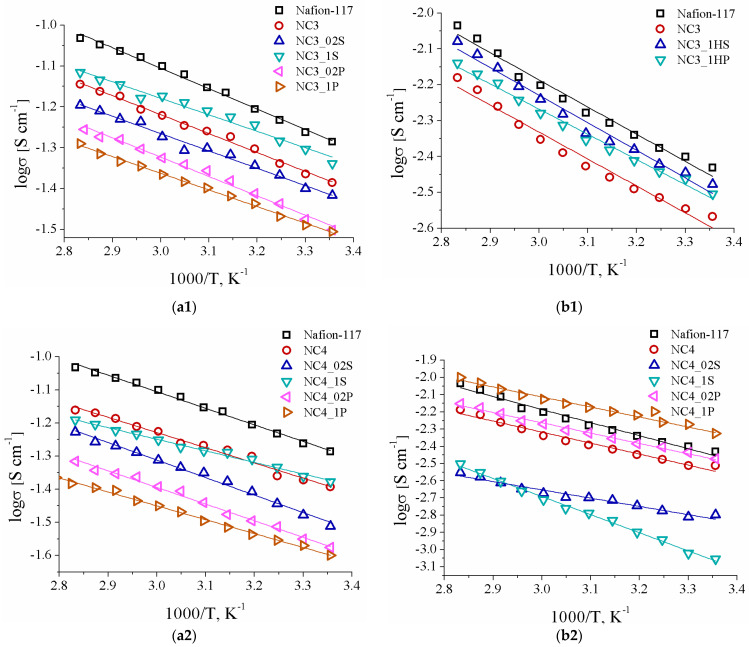

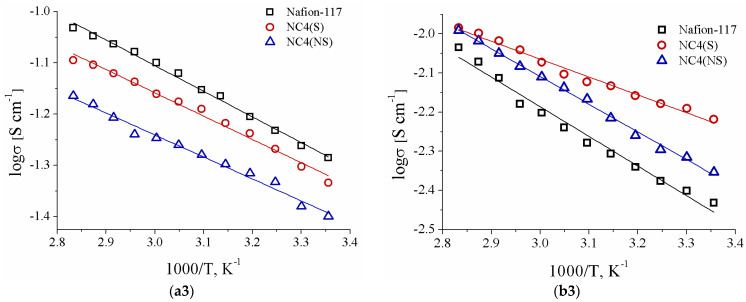
Temperature dependences of conductivity for the prepared hybrid membranes of the NC3 series (**a1**,**b1**), the NC4 series (**a2**,**b2**), the NC4(S) and NC4(NS) membranes (**a3**,**b3**) in contact with water (**a1**–**a3**) and at 30% relative humidity (**b1**–**b3**).

The conductivity of all the hybrid membranes in contact with water was lower than for the pristine Nafion-117 membrane (Figure 4(a1–a3)). The main reason for this is a decrease in the carrier concentration. Despite the higher water uptake, the conductivity of the NC4 series composite membranes was lower than that of the NC3 series samples. Most likely, this is due to the fact that cerium in the NC3 series samples blocks proton transfer only in the surface layer of membrane. For the membranes of both series, the conductivity of the hybrid membranes with ceria modified with phosphate groups was, in general, lower than the conductivity of the composite membranes with CeO_2_ modified with sulfonic acid groups, despite the fact that ceria modified by PO_4_-groups ex situ showed higher conductivity [41]. This may be due to the higher water uptake of the composite membranes with ceria modified by SO_4_-groups. In addition, phosphoric acid is significantly weaker than sulfuric acid. Thus, its dissociation in a membrane with high acidity is suppressed. Moreover, phosphate anions on the oxide surface can act as a "trap for protons", limiting their transport. The reason for the relatively low conductivity of NC4(NS) is apparently the partial substitution of protons for ammonium ions of the precursor.

The conductivity of all the hybrid membranes at 30% RH was almost an order of magnitude lower than the conductivity of these membranes in contact with water (Figure 4(b1–b3)), which is due to the partial membrane dehydration (Table 2). However, for the composite membranes with surface-modified ceria, this decrease was less pronounced. Moreover, the NC4(S), NC4(NS), and NC4_1P membranes exhibited higher conductivity compared with the pristine Nafion-117 membrane. It should be noted that this situation is typical for hybrid membranes [34].

**Figure 5:**
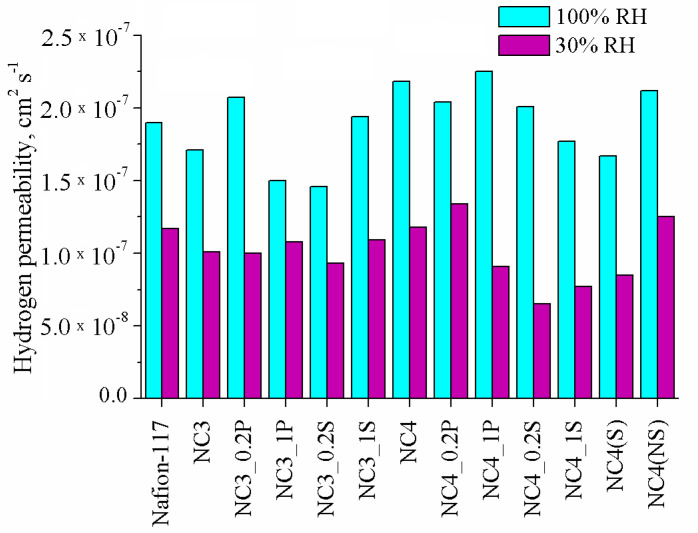
Hydrogen permeability at 30 and 100% RH of the studied composite membranes.

The hydrogen permeability of the composite membranes at 30 and 100% RH are shown in Figure 5. At 100% RH, the hydrogen permeability for most hybrid membranes turned was higher than in Nafion-117, with the exception of some samples of the Ce3 series. In this case, a significant ceria content in the near-surface membrane layers, as well as a lower water uptake of these membranes, prevented the hydrogen diffusion through the pore solution. With decreasing relative humidity, on the contrary, the hydrogen permeability for many hybrid membranes was lower. The reason for this is that at low RH, the membrane water uptake decreases and the contribution of the Debye layer, from which non-polar gas molecules are displaced, becomes increasingly significant.

Upon ceria incorporation, the mechanical properties of the hybrid membranes changed slightly (Figure S3). The Young modulus of the pristine Nafion-117 membrane was determined to be 257 ± 4 MPa. It increases slightly up to 260–275 MPa for the hybrid membranes with CeO_2_. Ceria incorporation seemed to increase the tensile strength slightly from 33.5 ± 0.4 MPa to 34–37 MPa (for the hybrid membranes). At the same time, the maximum strain for most membranes decreased slightly as compared with the pristine Nafion-117 (309 ± 3%). However, its magnitude did not decrease below 270%, and even increased to 312% for the NC4(NS) composite membrane. It can be assumed that a slight increase in Young’s modulus and tensile strength is determined by insignificant cross-linking of membranes upon dopant introduction, whereas its maximum strain also naturally decreases. We observed similar changes in the mechanical properties upon poly(3,4-ethylenedioxythiophene) introduction into the Nafion membranes [46]. It can be assumed that these changes, at least, will not lead to a deterioration in the mechanical stability of membranes in MEAs.

**Figure 6:**
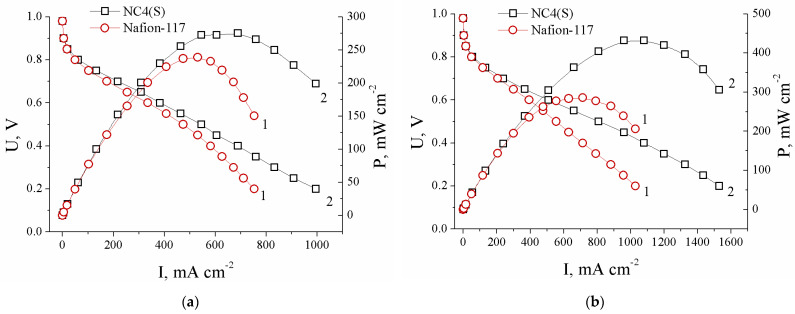
Polarization and power density curves for the MEAs with Nafion-117 (1) and the NC4(S) composite membrane (2) at 25 °C, 100% RH (**a**), and 60 °C, 70% RH (**b**).

As it is known, during the operation of low-temperature fuel cells, significant problems arise with membrane hydration, especially in the anode side, from which water molecules are partially transferred away along with protons. Therefore, it was of interest to test such membranes in fuel cells at a slightly reduced humidification. Current-voltage curves and dependences of power density on current density for MEAs with the Nafion-117 and NC4(S) membranes at 25 and 60 °C are shown in Figure 6. In the middle current range, the slope of the current-voltage curves of the MEAs based on the NC4(S) membrane was reduced compared with the MEAs with the Nafion-117 membrane, which indicates a decrease in the MEA internal resistance. Additionally, and which is not less important, the peak power density also significantly increased for MEAs with the modified membrane. Its values were 233 and 276 mW/cm^2^ at 25 °C and 287 and 433 mW/cm^2^ at 60 °C for the MEAs with Nafion-117 and the NC4(S) membrane, respectively. An increase in the current density at a voltage of 0.4 V corresponding to the dominance of the influence of ohmic and transport restrictions should also be noted. The MEAs based on Nafion-117 and the modified membrane delivered 582 and 689 mA/cm^2^ at 25 °C and 714 and 1080 mA/cm^2^ at 60 °C, respectively.

To test the ability of dopant to mitigate the membrane degradation, the pristine Nafion-117 and the NC4(S) composite membranes were treated with the Fenton solution (ex situ accelerated degradation test). The fluoride emission rate (the ratio between the mass of released fluoride ions and the initial membrane mass) was about four times lower for the NC4(S) membrane than for Nafion-117 (0.42 and 1.81 mg/g, respectively).

## 4. Conclusions

New composite membranes based on Nafion-117 and ceria with a surface modified by sulfonic acid or phosphate groups were prepared by the in situ method. When cerium (III) nitrate was used as a precursor, the dopant was concentrated in the near-surface layers of the composite membrane, while in the case of (NH_4_)_2_Ce(NO_3_)_6_, the dopant distribution over the membrane thickness was uniform. At low humidity, the proton conductivity of membranes with ceria modified with sulfonic acid groups exceeded that of the pristine Nafion-117 membrane. Hydrogen permeability decreased for almost all composite membranes with ceria obtained from cerium (III) nitrate, which correlates with their water uptake. Compared to the pristine Nafion-117 membrane, the peak power density of MEAs with the composite membrane containing ceria modified with sulfonic acid groups increased 1.5 times. Taking into account this and high stability of the hybrid membranes in the presence of peroxide moieties, it can be assumed that the prepared hybrid membranes have high potential for hydrogen-air fuel cell applications.

## Figures and Tables

**Table 1:** Manufactured hybrid membranes.

Sample	Ceria Precursor	Additional Treatment
Nafion-117	-	-
NC3	0.01 M Ce(NO_3_)_3_	-
NC3_0.2P	0.01 M Ce(NO_3_)_3_	0.2 M H_3_PO_4_
NC3_1P	0.01 M Ce(NO_3_)_3_	1 M H_3_PO_4_
NC3_0.2S	0.01 M Ce(NO_3_)_3_	0.2 M H_2_SO_4_
NC3_1S	0.01 M Ce(NO_3_)_3_	1 M H_2_SO_4_
NC4	0.3 M (NH_4_)_2_Ce(NO_3_)_6_	-
NC4_0.2P	0.3 M (NH_4_)_2_Ce(NO_3_)_6_	0.2 M H_3_PO_4_
NC4_1P	0.3 M (NH_4_)_2_Ce(NO_3_)_6_	1 M H_3_PO_4_
NC4_0.2S	0.3 M (NH_4_)_2_Ce(NO_3_)_6_	0.2 M H_2_SO_4_
NC4_1S	0.3 M (NH_4_)_2_Ce(NO_3_)_6_	1 M H_2_SO_4_
NC4(S)	0.05 M Ce(SO_4_)_2_	-
NC4(NS)	0.05 M (NH_4_)_4_Ce(SO_4_)_4_	-

**Table 2:** Dopant content (ω_dop_), ion-exchange capacity (IEC) and water uptake (ωH_2_O) at 95 and 30% RH of the prepared hybrid membranes.

Sample	ω_dop_, %	IEC ± 0.02, mmol/g	ωH_2_O, % (RH = 95%)	ωH_2_O, % (RH = 30%)
Nafion-117	-	0.91	23.2	5.3
NC3	2.2	0.80	23.7	4.7
NC3_0.2P	2.4	0.81	23.8	4.5
NC3_1P	1.8	0.83	23.9	4.5
NC3_0.2S	2.1	0.81	24.0	4.5
NC3_1S	1.3	0.84	24.2	4.5
NC4	0.8	0.85	26.2	4.9
NC4_0.2P	0.6	0.87	26.0	4.9
NC4_1P	0.5	0.88	26.3	5.0
NC4_0.2S	1.0	0.85	26.0	4.5
NC4_1S	0.9	0.87	26.9	4.4
NC4(S)	0.4	0.89	26.4	5.5
NC4(NS)	0.1	0.88	26.6	5.1

## Data Availability

The data presented in this study are available on request from the corresponding author.

## Supplementary Materials

The following are available online at https://www.mdpi.com/article/10.3390/polym13152513/s1, Figure S1: X-ray diffraction patterns of residues of the NC3 (1), NC3_1S (2), NC3_1P (3), NC4 (4), NC4_1S (5), and NC4_1P (6) hybrid membranes after annealing at 800 °C, Figure S2: Nyquist plots of the NC4(S) composite membrane recorded at different temperatures in contact with water (a) and 30% RH (b), Figure S3: Stress–strain responses of the pristine Nafion-117 (1) and the NC3 (2), NC4_1P (3), NC4(S) (4), and NC4(NS) (5) hybrid membranes.
