# Supplementary material for: Nafion/Surface Modified Ceria Hybrid Membranes for Fuel Cell Application
Source: Polymers (Basel). 2021 Jul 30;13(15):2513. doi: 10.3390/polym13152513 (PMC8348518; doi:10.3390/polym13152513)
Supplement: Supplementary file 1 [file polymers-13-02513-s001.zip › polymers-1275566-supplementary.pdf]

Supplementary information

(for article "Nafion/surface modified ceria hybrid membranes for proton exchange fuel cell application" by Polina A. Yurova, Viktoria R. Malakhova, Ekaterina V. Gerasimova, Irina A. Stenina and Andrey B. Yaroslavtsev)

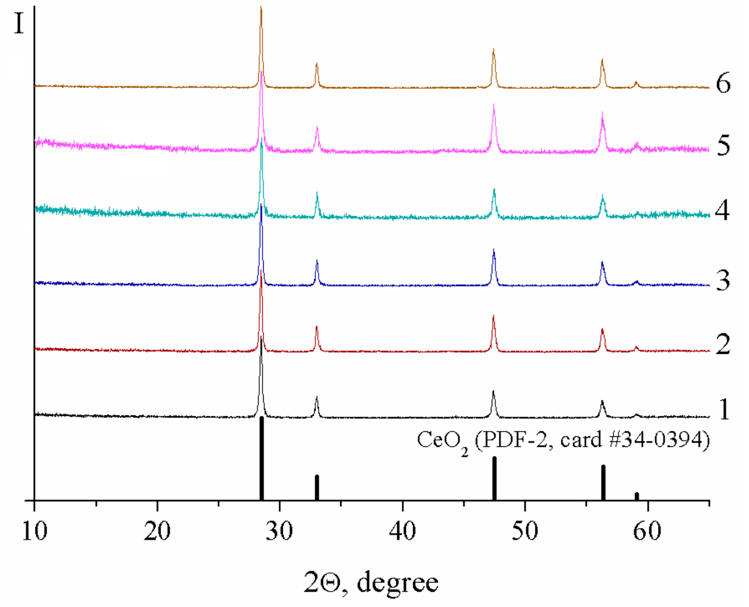

Figure S1. X-ray diffraction patterns of residues of the NC3 (1), NC3_1S (2), NC3_1P (3), NC4 (4), NC4_1S (5), and NC4_1P (6) hybrid membranes after annealing at 800 °C.

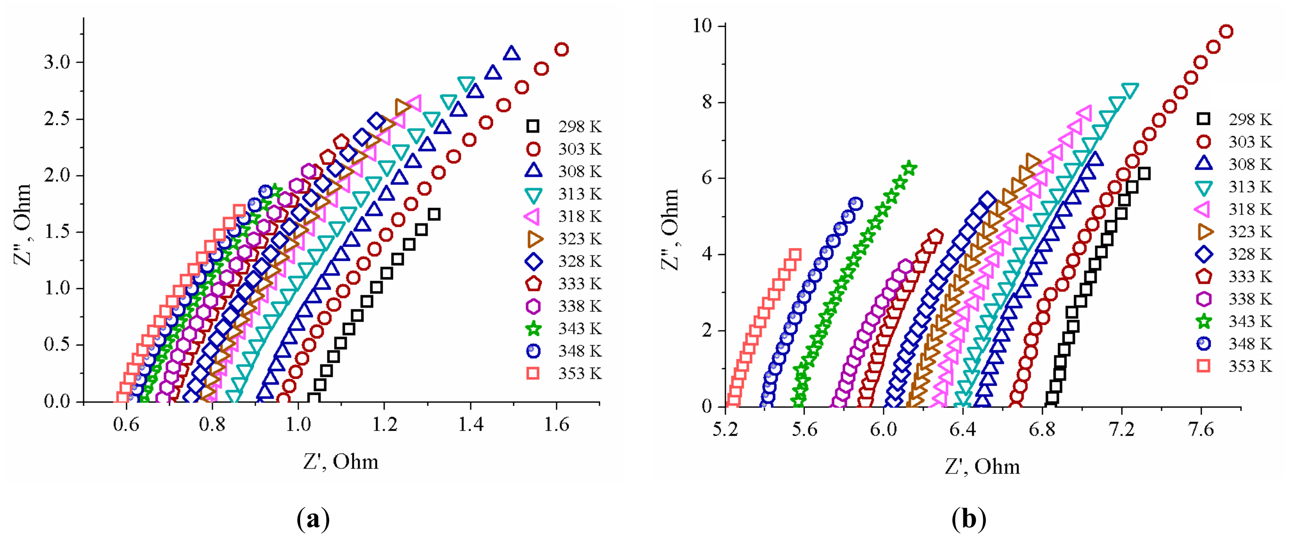

Figure S2. Nyquist plots of the NC4(S) composite membrane recorded at different temperatures in contact with water (a) and 30% RH (b).

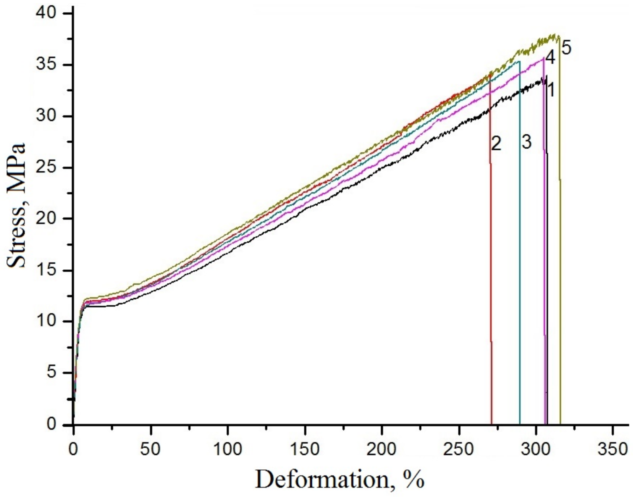

Figure S3. Stress–strain responses of the pristine Nafion-117 (1) and the NC3 (2), NC4_1P (3), NC4(S) (4), and NC4(NS) (5) hybrid membranes.
